# Magnetic resonance imaging histogram analysis of corpus callosum in a functional neurological disorder

**DOI:** 10.3906/sag-2004-252

**Published:** 2021-02-26

**Authors:** Sema BAYKARA, Murat BAYKARA, Osman MERMİ, Hanefi YILDIRIM, Murad ATMACA

**Affiliations:** 1 Department of Psychiatry, Faculty of Medicine, Fırat University, Elazığ Turkey; 2 Department of Radiology, Faculty of Medicine, Fırat University, Elazığ Turkey

**Keywords:** Conversion disorder, somatoform disorders, corpus callosum, histogram, magnetic resonance imaging

## Abstract

**Background/aim:**

The aim of the present study was to examine and compare the corpus callosum (CC) via histogram analysis (HA) on T1-weighted MR images of patients diagnosed with Functional Neurological Disorder (FND) and healthy controls.

**Materials and methods:**

The study group included 19 female patients diagnosed with FND, and the control group included 20 healthy subjects. All participants were scanned with a 1.5 T MR scanner. A high-resolution structural image of the entire brain was obtained with sagittal 3D spiral fast spin echo T1-weighted images. Gray level intensity, standard deviation of the histogram, entropy, uniformity, skewness, and kurtosis values were determined with texture analysis. A student’s t-test was used to compare the group data. P < 0.05 was accepted as statistically significant.

**Results:**

It was determined that the mean gray level intensity, standard deviation of the histogram, entropy calculated by the maximum, median and variance and size M percentage values were higher in patients with FND. Kurtosis and size U percentages values were lower in patients with FND.

**Conclusion:**

In the present study, analysis of CC with T1-weighted MR image HA demonstrated significant differences between FND patients and healthy controls. The study findings indicated that HA is a beneficial technique for demonstrating textural variations between the CCs of patients with FND and healthy controls using MR images.

## 1. Introduction

Functional neurological disorder (FND) is the presence of neurological symptoms in the absence of a neurological disease [1]. For diagnosis, the identified dysfunction should not be associated with a medical disorder [2]. The presence of one or more voluntary, motor, or sensory function changes and incompatibility and discrepancy between neurological or general medical conditions that may explain this appearance are included in the diagnostic criteria [2]. Although the findings reported in structural and functional brain imaging studies available in the literature did not sufficiently explain the physiopathology of FND [3], certain studies suggested and supported the presence of significant brain findings accompanying FND symptoms [1,3-8]. These findings have led to an emphasis on disorders associated with FND symptoms that could be identified with brain imaging and neurophysiological measurements [1,3–8].

The corpus callosum (CC) connects the cerebral hemispheres and contains the most nerve fiber tracts in the human brain. The main function of the CC is to connect the cerebral cortex regions to ensure interhemisphere integration. It acts as a transmission channel for transmitting sensory information [8,9]. It is known that CC agenesis is associated with symptoms of epilepsy, Asperger’s syndrome, learning disabilities, behavioral problems, depression, adjustment disorder, schizophrenia, and conversion disorder [10]. The presence of alexithymia, delusions, and hallucinations are shown in the presence of tumors in this region [11].

Diagnostic images used in clinical practice are digital. A two-dimensional digital image consists of small rectangular blocks called pixels (image elements). Each pixel is represented by a set of spatial coordinates and each has a value that represents gray level density in that image or volumetric element in space [12]. In the image, various mathematical methods used to determine spatial gray level variations in order to obtain derivatives called ‘texture properties’, which provide a measure of intralesional heterogeneity, are referred to as texture analysis. Texture analysis is basically a technique that analyzes signal properties, in other words, the position and density of the pixels and the gray level intensity in digital images [13]. Transform-based, structure-based, and statistics-based methods were developed to obtain textural properties using digital images [14]. Histogram-based texture analysis (HA) entails the calculation of all pixels of image densities in the region of interest (ROI) using a full statistical analysis [12]. Recently, these techniques have frequently been used to distinguish normal and abnormal structures [13], characterize tumors [15], guide radiotherapeutic treatment [16], and identify various biomarkers [17–19].

Studies in the literature have shown that either the congenital abnormalities or degenerative processes (due to tumors, traumas, hydrocephalus, vascular accidents, and white matter diseases) of the CC, which also serves as a conduit for transfer of sensory information, were related to conversion symptoms [20]. Based on this information, the present study aimed to assess the CC through HA using T1-weighted MR images of patients with FND and to compare the findings with healthy controls.

## 2. Materials and methods

### 2.1. Study population

After Local Ethics Committee approval, 20 patients, who were diagnosed with FND based on DSM-5 criteria in the psychiatry outpatient clinic, were included in the study. Since one of the patients was not considered suitable for magnetic resonance (MR) imaging (MRI) analysis, the study group included 19 patients. All patients who volunteered to participate in the study were female and had psychogenic nonepileptic seizures as leading symptom. Their neurological examinations (including electroencephalogram and MRI analyses) were made by neurology specialists before admission to the psychiatry clinic. They were referred to the psychiatry outpatient clinic by neurology specialists after a diagnosis of epilepsy was excluded. FND diagnosis was confirmed by a psychiatry specialist through a secondary evaluation. All the participants were on antidepressants, including selective serotonin reuptake inhibitors or tricyclic antidepressants (Imipramine and Clomipramine) at least for the last 3 months. Drug doses were stabilized 1 month before the study. Study criteria included patients being between 18–65 years of age, the presence of no other psychiatric diagnosis, no mental retardation, no history of neurological or physiological disease or disease history, no head trauma history, no alcohol or substance abuse history during the last 6 months, and no contraindication for MRI examinations. All subjects completed a sociodemographic and clinical data form developed by the authors based on their clinical experiences and information obtained with a literature review and the study objectives. The semistructured form included sociodemographic information such as age, sex, marital status, educational level, and occupation; clinical information such as age at the onset of the disease, duration of the disease, and the presence of another comorbid disease was also included. Patients with comorbid psychiatric and physiological disorders were excluded based on patient statements and the information obtained from the hospital information system. 

Healthy individuals who met the study criteria and matched with the patient groups based on age and sex, who were not diagnosed with a psychiatric, neurological, or physiological disease, who had no history of medical treatment in the last 3 months, and who had no contraindications for MRI examinations were included in the control group (20 individuals). The MRI was conducted after all participants signed an informed consent form that reflects the methodology and the aim of the study in detail.

### 2.2. MRI exam

For the MRI, a 1.5 Tesla GE SignaHDxt (GE Medical System, Milwaukee, WI, USA) scanner with an 8-channel HR brain coil and CUBE sequence was used. T1-weighted sagittal 3D fast spin echo high resolution MRI images were obtained. MRI parameters were repetition time [TR]: 600 ms; echo time [TE]: 10.178 ms; field of view [FOV]: 240 mm; slice thickness/spacing: 1/0.5 mm; number of excitations = 1; echo train length: 28; and matrix size: 256 × 256.

### 2.3. Image analysis

The gray level density histogram is a concise and simple summary of the statistical information included in the image. The calculation of the gray level histogram includes the individual pixels. Thus, the histogram provides first-degree statistical information about the image [12]. This information includes the mean gray level intensity, standard deviation of the histogram, median, minimum and maximum intensity values, variance values, entropy, uniformity, skewness, kurtosis, and size % lower, size % upper, size % mean (size % L, size % U, and size % M) values.

Entropy is a texture analysis parameter and known to measure parametric homogeneity within the ROI [21,22]. This is a measure of gray level variation in a histogram and is a parameter that indicates inhomogeneity (irregularity) in the intensity. When all data are the same, it is defined as zero, and the value increases as the distribution becomes irregular [22,23].

Uniformity refers to the gray level distribution, which indicates the closeness of the gray tones of the image to uniform distribution; a higher figure implies a more uniform distribution [22,23]. Skewness reflects the asymmetry of the distribution; if there are more points on the left side of the mean, the skewness is positive and negative when the opposite is true [23]. Kurtosis is a measure of the peak of distribution. If the histogram is a bell curve, kurtosis is 3, and if the histogram has a sharper peak, it is greater than 3 [23]. Size % L, size % U, and size % M depict the area between ±1 standard deviation and the histogram curve (size % M), the area above +1 standard deviation (size % U), and the area below –1 standard deviation (size % L) [24]. Images were transferred to a 27-inch iMac computer (Apple Inc., Cupertino, CA, USA). OsiriX V.4.9 imaging software (Pixmeo, Switzerland) was used to conduct the HA on the ROI.

**Figure 1:**
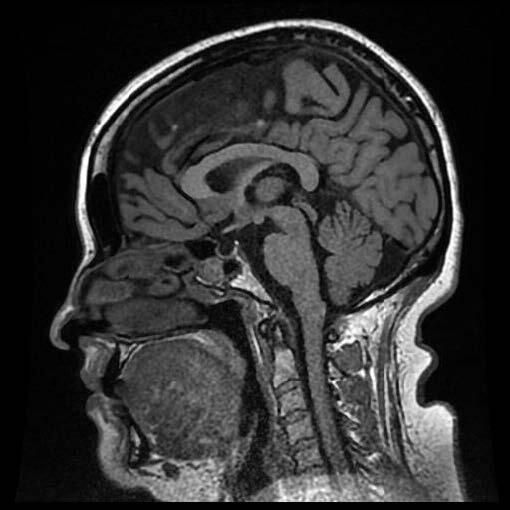

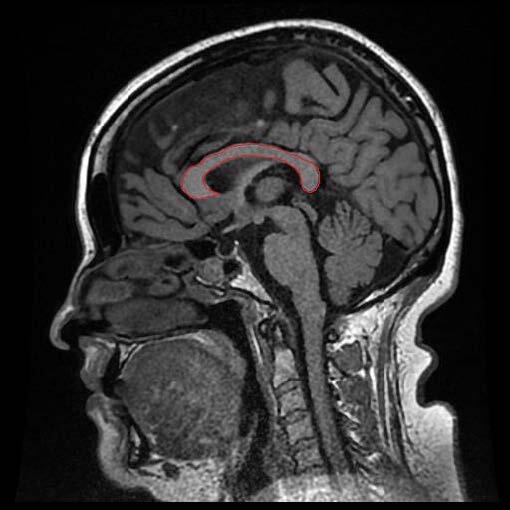
Selected section for ROI (A) and marked ROI (B) of a patient.

The ROI included the entire CC region in the midsagittal section because it is best seen and demarcated there (Figure 1). Gray level intensity, standard deviation of the histogram, entropy, uniformity, skewness, kurtosis, and size % L, size % U, and size % M values were calculated using the ROI values. The entire image analysis algorithm was provided by software developed in-house with MATLAB (version R2009b; MathWorks, Natick, MA, USA).

### 2.4. Statistical analysis

Data were expressed as mean ± standard deviation. According to normal distribution analysis (One Sample Kolmogorov–Smirnov test) of variables, a Student’s t-test and a Mann–Whitney U test were used to compare the groups. All statistical analyses were made with IBM SPSS for Windows, version 22.0 (Windows v. 22, IBM Corp., Armonk, NY, USA). All probability values were two-tailed. A value of P < 0.05 was considered statistically significant.

## 3. Results

**Figure 2:**
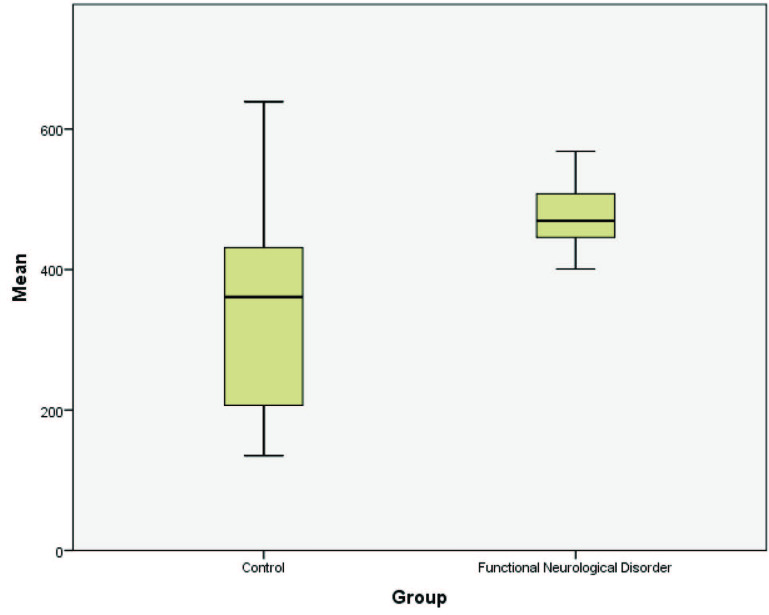
Mean gray level intensity value distribution of the groups.

**Figure 3:**
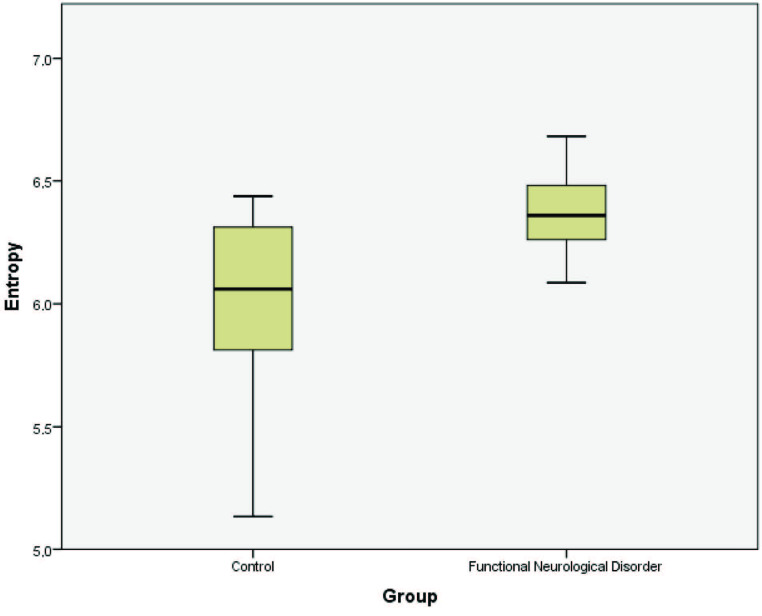
Entropy value distribution of the groups.

All patients and control subjects were female. The mean age was 33.79 ± 9.97 in the patient group and 32.745 ± 8.50 in the control group. There was no significant difference between the groups based on age (P = 0.728). There was no significant difference between the CC regions in the crosssectional images of the patients diagnosed with FND (P = 0.105). The mean gray level intensity, standard deviation of the histogram, maximum, median and variance values of entropy, and size % M were higher in patients with FND when compared to the control. The analysis of histogram parameters of the groups is shown in the Table. The mean gray level intensity is shown in Figure 2. Kurtosis and size % U values were lower in patients with FND when compared to the control. Entropy value distribution of the groups is shown in Figure 3.

**Table:** Analysis of histogram parameters of the groups

	Control	Functional neurological disorder	P
	Mean ± standard deviation	Mean ± standard deviation	
Mean	336.56 ± 147.21	476.10 ± 46.09	<0.001
Standard deviation	31.26 ± 13.76	75.49 ± 11.47	<0.001
Minimum	115.55 ± 77.96	80.20 ± 40.30	0.080
Maximum	402.55 ± 170.54	596.50 ± 59.41	<0.001
Median	342.20 ± 148.22	500.60 ± 41.92	<0.001
Variance	1157.38 ± 989.52	5823.68 ± 1704.56	<0.001
Entropy	6.01 ± 0.35	6.38 ± 0.22	<0.001
Size %Lower	9.55 ± 1.77	10.65 ± 3.09	0.072
Size %Upper	6.76 ± 3.15	2.69 ± 2.33	<0.001
Size %Mean	83.69 ± 3.50	86.66 ± 2.56	0.004
Kurtosis	13.80 ± 4.34	10.34 ± 2.89	0.006
Skewness	-2.50 ± 0.46	-2.58 ± 0.47	0.611
Uniformity	0.61 ± 0.05	0.63 ± 0.05	0.073
Area (cm2)	5.64 ± 0.69	6.07 ± 0.94	0.105

## 4. Discussion

FND diagnosis is based on evidence that the existing symptoms (sensory, motor, and neurovegetative) are incompatible with any known neurological disease [25]. Since the 19th century, it has been established that there was no anatomic and functional pathology in the brains of patients with conversion disorder, and the absence of abnormal findings in imaging results was a supportive factor in the diagnosis of FND for the clinician. In recent years, however, study findings have indicated structural and functional changes in regions of the brain associated with the symptoms [1,3–8,26–28]. The CC provides integration between the hemispheres by connecting related cerebral cortex regions in the brain [8,9]. It was demonstrated that disruption of the interhemispheric connectivity leads to a decrease in the transfer of learned information from one hemisphere to the other, the impairment of the visual pattern matching between the right and left visual fields, and symptoms of delusion, hallucination, and alexithymia [29]. It is known that alexithymia is also prevalent in patients with FND [30]. Schizophrenia, Asperger’s syndrome, personality disorder, depressive symptoms, and conversion symptoms have been shown in patients with CC agenesis [29]. Examining the white matter, including CC and its relation with psychogenic states was previously examined in the literature in many other studies [31–34]. In the study conducted by Huber et al., white matter structures were related to mood by showing that emotional stimulation showed activation of superior and inferior frontal gyri, as well as the CC [31]. Moreover, it was shown that in humans with depression, white matter lesions occurred, and the size and metabolic rate of the CC were modified [32–34]. Another study showed volumetric reductions of the genu and isthmus regions of the CC in euthymic bipolar patients with a history of suicide attempts [35]. Empirical evidence from neuroimaging research and from noninvasive brain stimulation studies suggests that the CC plays a significant role in mood states [36]. However, structural neuroimaging is not able to extract information on the functional status of the CC [37]. Texture analysis identifies structures that could not be visually assessed based on the description of microstructural information and provides more valuable information about structural patterns when compared to shape-based measurements [38]. Mostly, it allows for the classification of the tissue as pathological or healthy based on ROI images or by distinguishing various anatomical structures. Recently, assessments of MR images using texture analysis or HA methods have become a focus of interest in the investigation of the etiology of psychiatric disorders [39,40]. In a study, in which brain MRIs of schizophrenia patients and healthy controls were investigated with texture analysis, it was demonstrated that the entropy and uniformity of gray matter of the patients with schizophrenia were significantly different when compared to that of the healthy controls. The study findings emphasized the abnormal distribution of gray matter and focal structure of the hippocampus in schizophrenia patients [38]. In the literature review, it was observed that CC has not been analyzed with histogram-based texture analysis in patients with FND before. In the present study, a statistical approach was adopted to determine the texture information in the images based on the gray level distribution of the pixels. This method provides better results with medical imaging when compared to older methods such as structural approaches [14]. 

The participants in the study were on antidepressants, including selective serotonin reuptake inhibitors or tricyclic antidepressants (Imipramine and Clomipramine) at least for the last 3 months. Reyes-Haro et al. established the existence of 5-HT uptake into an adult rat CC and that this uptake was partially inhibited by antidepressant drugs including fluoxetine, imipramine, and clomipramine [41]. However, the effects of antidepressants on CC are not well known. We will have more data on the issue with further studies about the effects of the antidepressants on the CC.

In the present study, although there was no significant difference between the CC regions of the FND patients and controls in the crosssectional images, mean gray level intensity, standard deviation of the histogram, entropy, maximum, median and variance values, and size % M values were higher in the FND cases, while size % U values were lower in patients with FND. Entropy and homogeneity represent additional imaging parameters that could measure homogeneity and brightness. Entropy is a measure of the distribution of elements in an image [42]. In the present study, the fact that entropy, standard deviation, and variance values were high indicates that the distribution within the ROI became increasingly irregular [13]. It was determined in the present study that the CC MRIs of patients with FND were different when compared to that of the healthy controls when examined via HA. It could be suggested that the distribution of the CC image was irregular, and this irregularity increased in FND cases. 

The present study had certain limitations. Since there were no previous studies that analyzed the CC with HA in patients with FND, we were not able to compare the study findings. The fact that all of our patients were female prevented the generalization of the study findings for both sexes. The small sample size of patients and controls was another limitation of the study.

## 5. Conclusion

In the present study, the analysis of the CC with HA using T1-weighted MR images revealed significant differences between patients with FND and healthy controls. The study findings indicated that HA is a beneficial technique for demonstrating tissue changes between the CCs using the MR images of patients with FND and healthy controls. Future studies with larger samples may contribute to further explanation of these findings. The present study may help future researchers to further elucidate the etiology of FND, its diagnosis, and the determination of treatment options.
